# Modeling and Forecasting the Operation of the Emergency Medical Care System in the Republic of Kazakhstan

**DOI:** 10.3390/healthcare14162540

**Published:** 2026-08-14

**Authors:** Gulnara Batenova, Diana Ygiyeva, Zhanar Urazalina, Andrey Orekhov, Akbayan Markabayeva, Maksim Pivin, Adilzhan Zhumagaliyev, Lyudmila Pivina

**Affiliations:** 1 Department of Emergency Medicine, Semey Medical University, Semey 071400, Kazakhstanzhumagaliev.2002@mail.ru (A.Z.);; 2 Department of Internal Medicine, Semey Medical University, Semey 071400, Kazakhstan; 3 Department of Family Medicine, Astana Medical University, Astana 010000, Kazakhstan; 4 Medical Center Hospital of the President’s Affairs Administration of the Republic of Kazakhstan, Astana 010000, Kazakhstan; pivin97@mail.ru

**Keywords:** health workforce planning, healthcare forecasting, time series analysis, health services research, emergency care system

## Abstract

**Background/Objectives:** Emergency medical care is a critical component of the healthcare system. The aim of the study was to forecast the number of general practitioner and paramedic teams, the number of ambulance stations, and the number of calls per 1000 population in Kazakhstan until 2030. **Methods:** We conducted a retrospective longitudinal ecological time-series study of the staffing and structure of the emergency medical service in Kazakhstan for 2000–2023. Data were provided by emergency medical stations in 16 regions of Kazakhstan. We also used data from the statistical digest “Health of the Population of the Republic of Kazakhstan and the Activities of Healthcare Organizations”. **Results:** A progressive decrease in the absolute number of emergency medical system (EMS) stations by an average of 4.19% (95% CI: [−5.00%, −3.37%]) was found. In the period from 2000 to 2023, a relative average annual decrease was found in the number of medical teams by 7.94% [CI −10.14%, 5.69%] and pediatric teams by 7.73% [CI −10.36%, 5.02%]. An analysis showed an average annual growth rate of paramedic teams of 1.62% [CI 0.19%, 3.46%]. The construction of a predictive model for the number of medical and pediatric teams and EMS stations in the country for the coming years demonstrates a further gradual decrease in their number. **Conclusions:** A predictive model for the number of physician teams predicts a further decline through 2030, while the forecast for the number of paramedic teams remains relatively stable.

## 1. Introduction

Emergency medical care is a critical component of the healthcare system, especially in low- and middle-income countries. The effectiveness and timeliness of emergency medical care depend on the availability of resources, primarily competent medical personnel. A shortage of medical teams and ambulance stations can lead to the collapse of the healthcare system in emergency situations such as natural disasters, infectious disease epidemics, military operations, or accidents [1,2,3]. This problem is especially pressing in the context of political or military instability. In the Republic of Kazakhstan, the shortage of ambulance and urgent medical care teams is due to the country’s large territory with a very low population density, which leads to prolonged response times, harsh climatic conditions with a wide range of temperatures, underdeveloped infrastructure, especially in rural areas, and insufficient use of digital technologies in emergency medical services [4,5,6,7].

The emergency medical service in Kazakhstan is coordinated by the National Coordination Center for Emergency Medicine, which provides emergency and urgent medical care to patients in the form of air ambulances and regulates the work of regional ambulance stations throughout the country [8]. At the regional level, the emergency medical service includes ambulance stations with the necessary medical equipment, ambulance physician and paramedic teams, and dispatch centers. The stations are uniform in design and technical characteristics; they provide access to medical information systems at various levels of the organization and ensure integration with medical information systems for the coordinating organization [9]. Unlike some European countries and the United States, where emergency medical care is provided in a unified manner by a single organization [10,11,12], in the Republic of Kazakhstan, the quality and availability of medical care may vary in different regions due to the low population density, different climatic conditions, and large areas of the regions. Therefore, the Ministry of Health of the Republic of Kazakhstan regularly adopts regulatory legislative acts to improve the regulation and management of emergency medical services in all regions of the republic [6].

The aim of the study was to forecast the number of physician and paramedic teams, the number of ambulance stations, and the number of calls per 1000 population in the Republic of Kazakhstan until 2030.

## 2. Materials and Methods

### 2.1. Data Collection

This study was a retrospective longitudinal ecological time-series study based on annual population-level data from 2000 to 2023. Historical trends were analyzed, and future tendencies were projected through 2030. The study was based on annual official aggregated statistics obtained from EMS stations in all 16 administrative regions of Kazakhstan and the national statistical digest Health of the Population of the Republic of Kazakhstan and the Activities of Healthcare Organizations. Complete annual data were available for the entire study period (2000–2023), and no missing years or imputed values were identified. The analysis was performed using officially reported national aggregate data; therefore, no additional procedures for data imputation or correction were required. The definitions of EMS stations, medical teams, and paramedic teams were based on the official reporting standards of the Ministry of Health of the Republic of Kazakhstan applicable during each reporting year. Although organizational reforms and regulatory changes occurred during the study period, these were considered part of the evolution of the national EMS. Considering that Kazakhstan is a unitary state, all regions have uniform approaches to the organization of the healthcare system, determined by the Ministry of Health of the Republic of Kazakhstan, uniform reporting forms and classifications.

### 2.2. Statistical Analysis

Statistical analysis of the data was performed using SPSS version 20.0 (BM SPSS Statistics for Windows, Version 20.0 (IBM Corp., Armonk, NY, USA)). Friedman’s two-way analysis of variance by ranks was used to compare key ambulance service performance indicators across years for related samples. Pillai’s trace test was used to analyze trends across years. To visualize the dynamics of the number of ambulance stations, the number of calls made, and the number of teams, we constructed time series for the period from 2000 to 2023. WinPepi was used to calculate the average annual growth or decline rate of the number of ambulance stations, the number of calls made, and the number of ambulance teams based on values for a given time period. In addition, 95% confidence intervals were also calculated for these indicators.

Temporal trends were assessed with the Joinpoint Regression Program version 6.1.0 (National Cancer Institute, Bethesda, MD, USA) using a log-linear regression model. The minimum and maximum numbers of breakpoints were set to 0 and 1, respectively. Annual percentage changes (APCs), 95% confidence intervals (95% CI), and *p*-values were calculated for each segment. A scenario forecast for 2024–2030 was then generated in R version 4.5.1 (R Foundation for Statistical Computing, Vienna, Austria) by extrapolating the last Joinpoint segment. To quantify the uncertainty of the forecast, 80% and 95% confidence intervals were calculated.

## 3. Results

**Figure 1:**
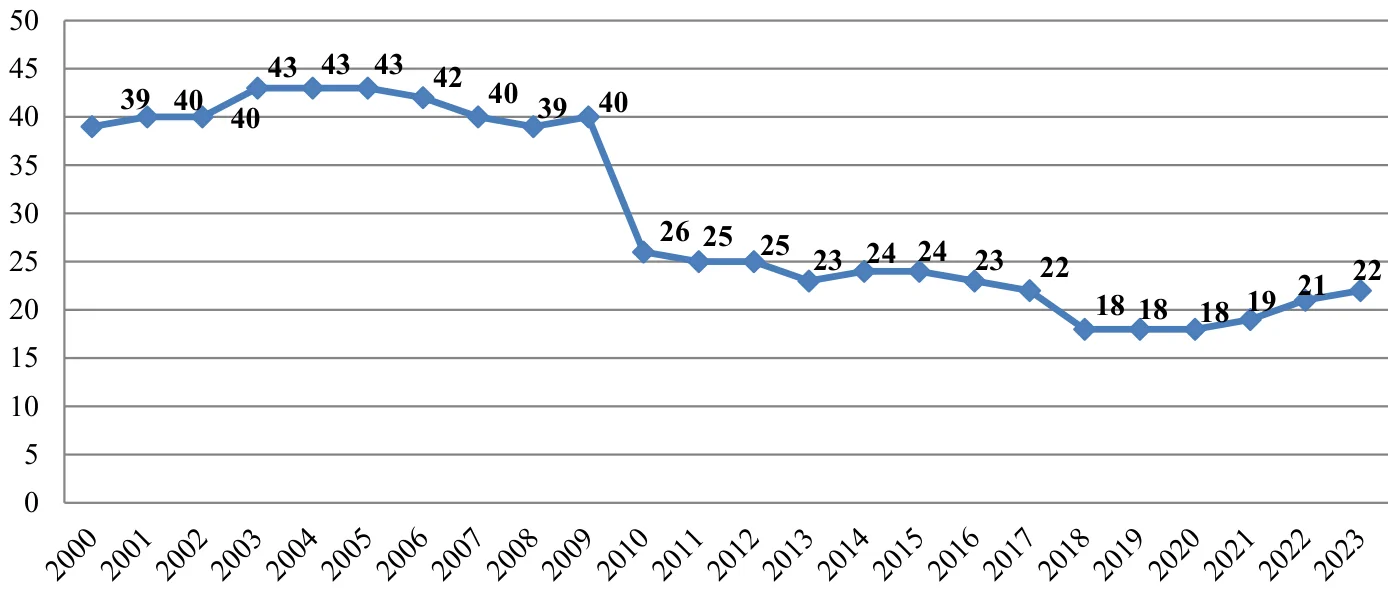
The number of EMS stations in the Kazakhstan in dynamics in the period 2000–2023.

An analysis of the number of EMS stations in dynamics from 2000 to 2023 in the Republic of Kazakhstan demonstrates a progressive decrease in the absolute number of independent EMS stations by an average of 4.19% (95% CI: [−5.00%, −3.37%]) (Figure 1). From 2000 to 2010, the number of stations remained stable, with a tendency to increase from 39 to 43 in 2003–2007. A sharp decrease in the number of EMS stations was noted in 2010—from 40 to 26 stations (the average annual rate of decline from 2009 to 2017 was 4.69%, CI [−8.36; −0.87]).

**Figure 2:**
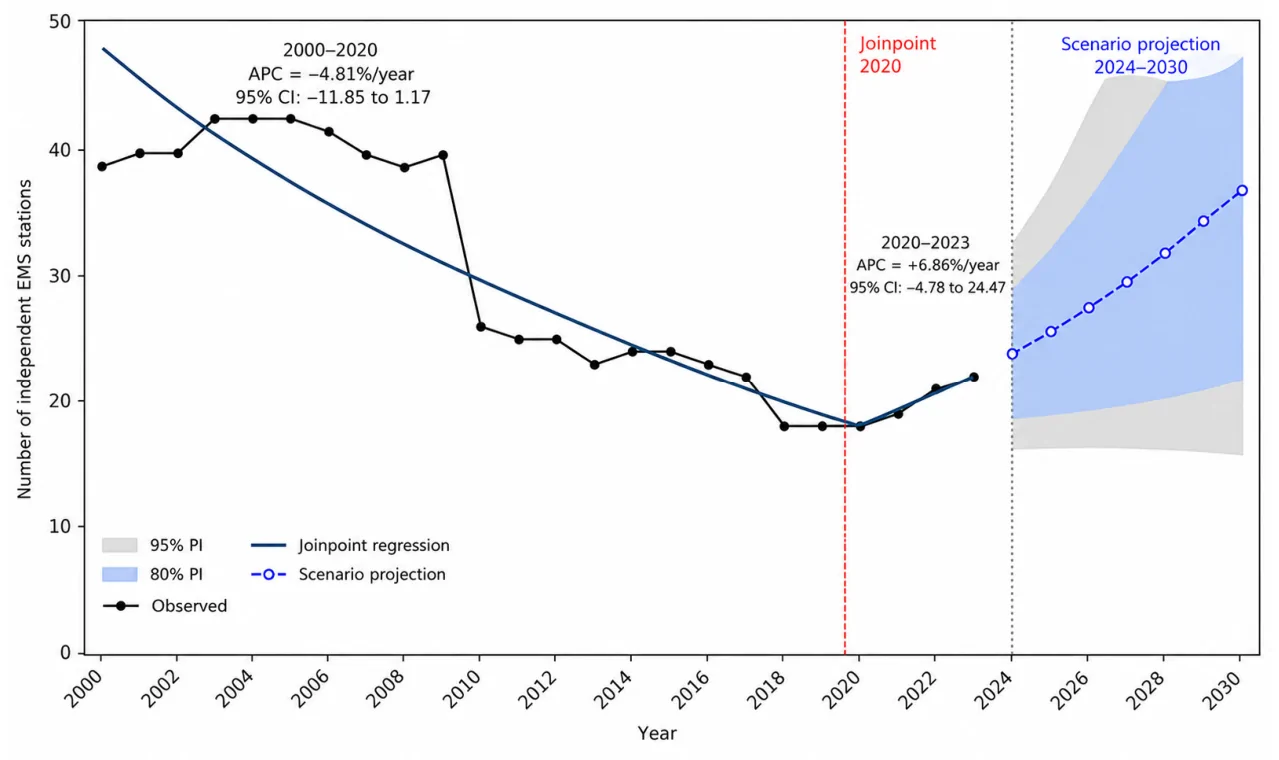
Observed time trends in the number of ambulance stations in Kazakhstan from 2000 to 2023, the resulting Joinpoint regression model, and a scenario forecast to 2030. The vertical dotted line represents the estimated Joinpoint (2020). The blue dotted line represents the extrapolation of the final Joinpoint segment. The shaded areas indicate the 80% and 95% confidence intervals of the forecast.

Regression analysis using the Joinpoint method identified one change point in the time trend of the number of ambulance stations in Kazakhstan in 2020 (95% CI: 2002–2021). During the first period (2000–2020), the number of ambulance stations showed a decreasing trend with an average annual percentage change (APC) of −4.81% (95% CI: −11.85 to 1.17; *p* = 0.076). During the second period (2020–2023), the trend changed towards a moderate increase (APC = 6.86%; 95% CI: −4.78 to 24.47; *p* = 0.527), although this increase was not statistically significant (Figure 2). Based on the extrapolation of the final Joinpoint segment, the scenario forecast assumes an increase in the number of independent ambulance stations from 23.9 in 2024 to 37.1 by 2030. However, the forecast intervals have widened significantly over time (95% confidence intervals: 16.3–35.0 in 2024 and 16.2–84.9 in 2030), indicating significant uncertainty. Therefore, these estimates should be interpreted as scenario forecasts rather than exact predictions. 

**Figure 3:**
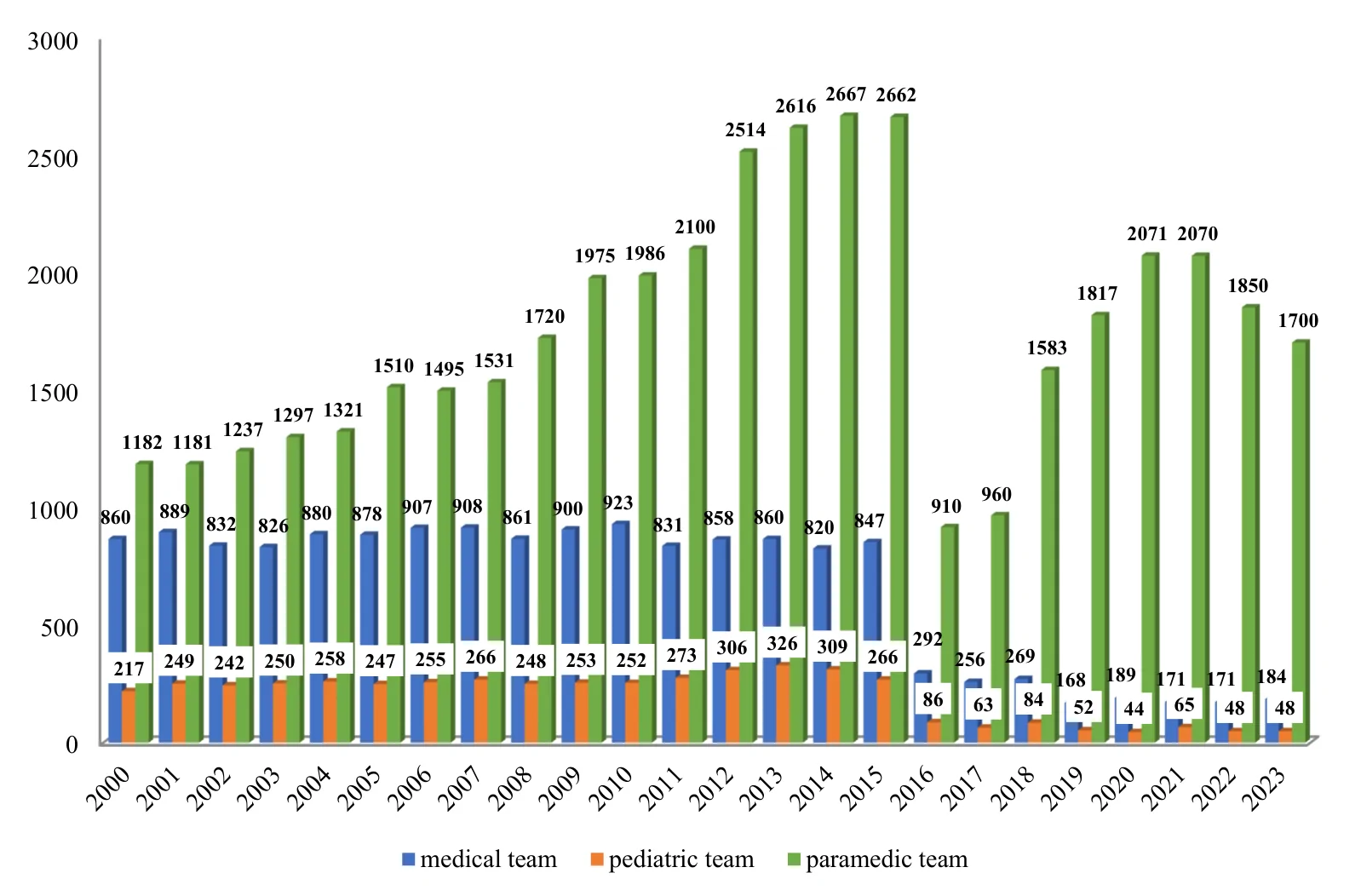
Dynamics of the number of general and paramedic teams in the period 2000–2023.

We were interested in analyzing the number of medical and paramedic teams in the Republic of Kazakhstan over the studied period, taking into account the increase in calls and the decrease in the number of EMS stations. In the period from 2000 to 2023, an analysis of the dynamics of the number of medical and pediatric teams in the Republic of Kazakhstan showed a relative average annual decrease in the number of medical teams by 7.94% [CI −10.14%, 5.69%] and pediatric teams by 7.73% [CI −10.36%, 5.02%]. As for paramedic teams, an analysis of the dynamics of the number of these teams in the Republic of Kazakhstan showed an average annual growth rate of paramedic teams of 1.62% [CI 0.19%, 3.46%]. While in 2000, the number of medical teams, including pediatric teams, was comparable to the number of paramedic teams (1077 vs. 1182), subsequently, there was an annual decline in the number of medical and pediatric teams compared to paramedic teams. Thus, in 2010, the number of general medical teams was 1175, while the number of paramedic teams was 1986 (37% of medical teams). In 2015, the number of general medical teams was 1113, while the number of paramedic teams peaked at 2662 (the share of medical teams fell to 29%). In 2016, there was an even more pronounced decline in the number of general medical teams to 378, while the number of paramedic teams fell to 910 (29% of medical teams). In 2020, at the onset of the coronavirus pandemic, the number of general medical teams fell to 233, while the number of paramedic teams increased to 2071, with the share of general medical teams in the overall structure being 10.1%. Until 2023, the number of medical and pediatric teams remained at the same level—232—while the number of paramedic teams decreased to 1700, and the share of medical teams was 12% (Figure 3).

**Figure 4:**
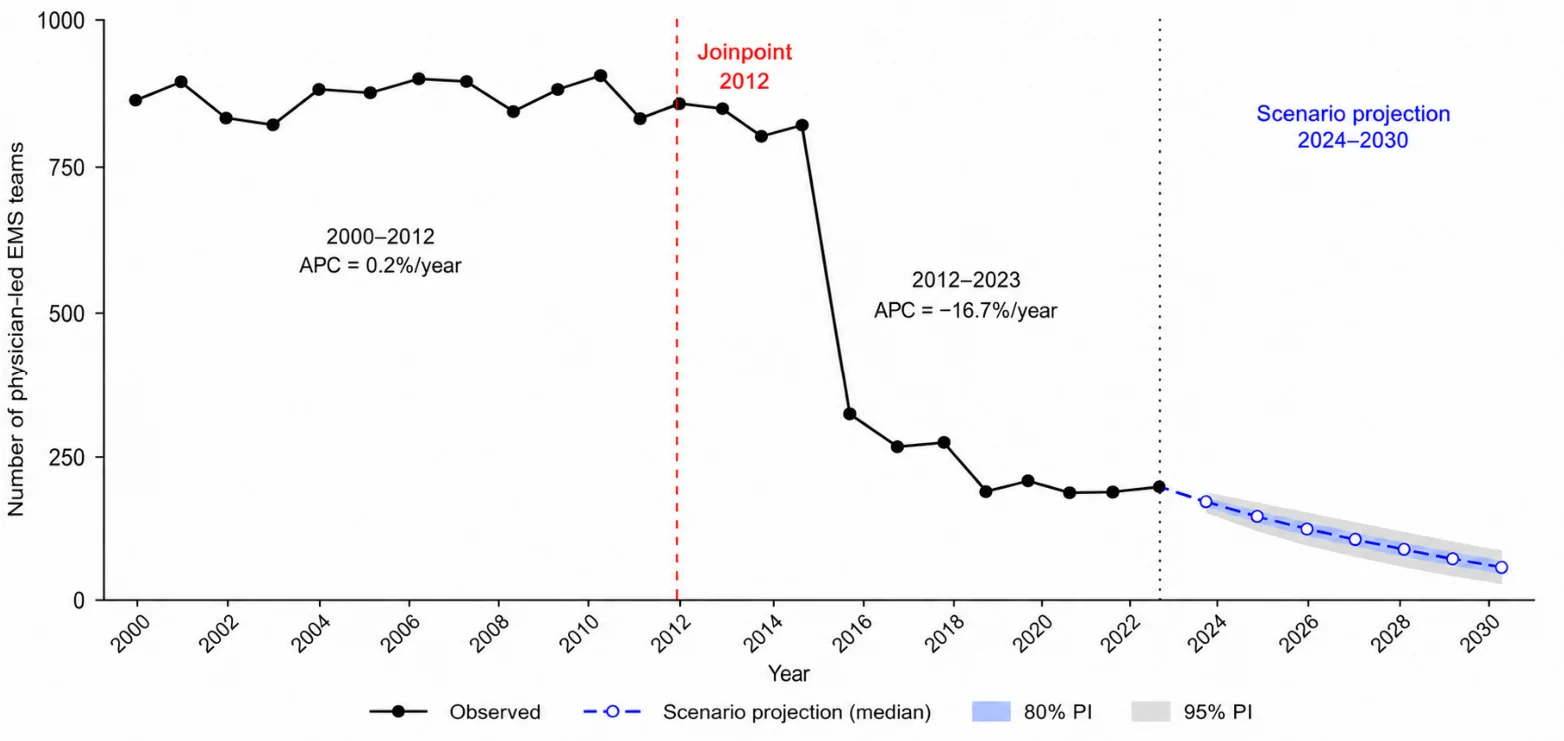
Observed trends (2000–2023), Joinpoint analysis, and scenario forecast (2024–2030) for the number of EMS medical teams.

The dynamics of the number of physician-led emergency medical teams is described by a Joinpoint regression model with a single change point identified in 2012 (Figure 4). From 2000 to 2012, the number of such emergency medical teams remained relatively stable (CAGR = 0.2%). After 2012, the temporal pattern changed significantly, with a steady decline observed from 2012 to 2023 (CAGR = −16.7%). The most pronounced decline occurred between 2015 and 2016, after which the number of physician-led teams stabilized at a significantly lower level. A forecast scenario for 2024–2030 was then created in R by extrapolating the final Joinpoint segment. The results suggest that the number of physician-led ambulance crews will gradually decline until 2030. However, the forecast intervals gradually widen with increasing forecast horizon, indicating significant uncertainty associated with long-term scenario estimates. Therefore, these values should be interpreted as scenario-based forecasts rather than exact predictions.

**Figure 5:**
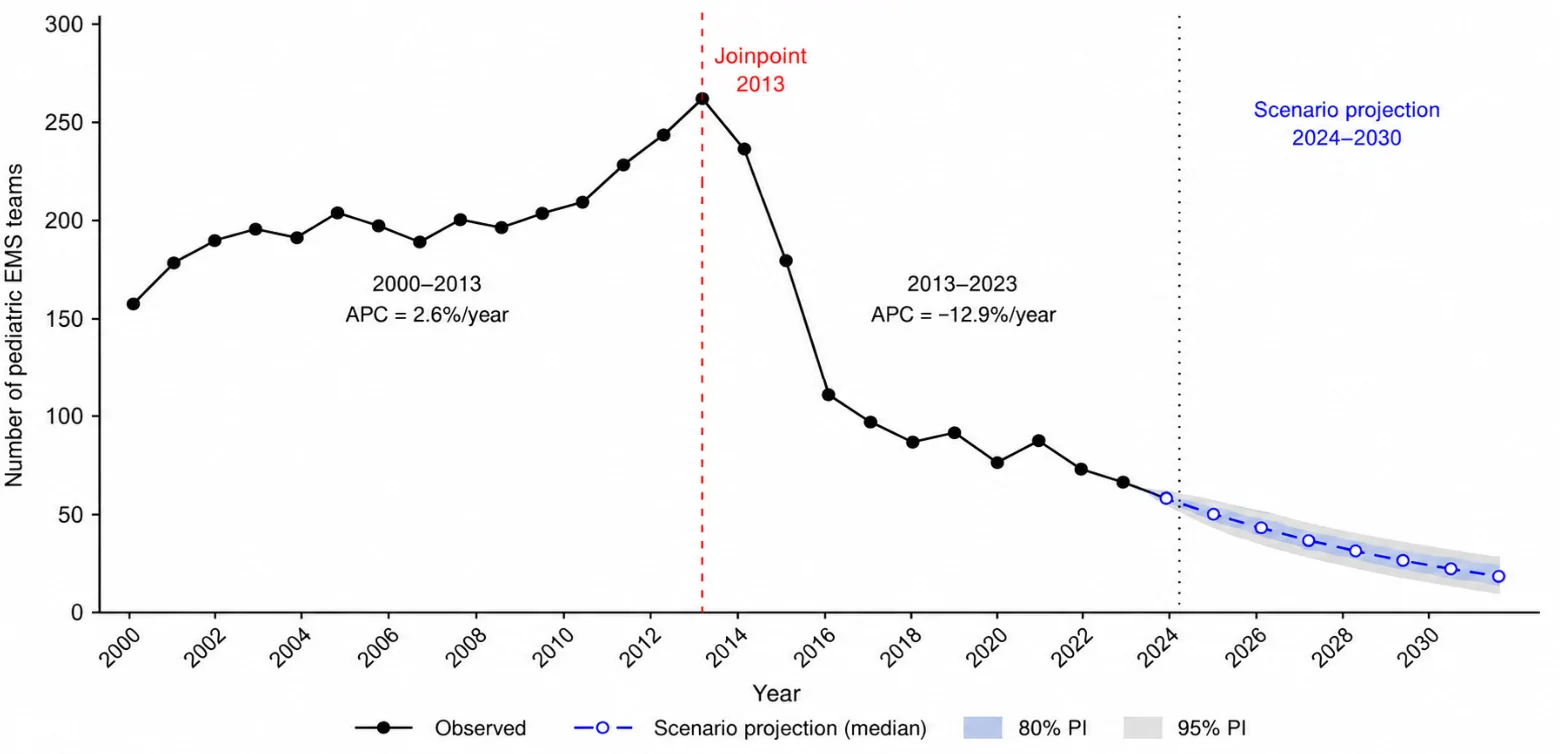
Dynamics of the number of pediatric EMS teams in 2000–2023, results of Joinpoint analysis and scenario forecast for 2024–2030.

The resulting Joinpoint regression model assessing the dynamics of pediatric EMS team numbers revealed a single change point in 2013 (Figure 5). Between 2000 and 2013, the number of these EMS teams demonstrated a moderate upward trend (CAGR = 2.6%). After identifying the change point, the temporal pattern changed significantly, with a steady decline observed between 2013 and 2023 (CAGR = −12.9%). The most pronounced decline occurred between 2015 and 2016, after which the number of pediatric EMS teams remained relatively stable at a significantly lower level. A forecast scenario for 2024–2030 was then created in R by extrapolating the final changepoint segment. Assuming the current trend continues, a gradual decline in the number of pediatric emergency medical teams is projected through 2030. The forecast intervals gradually widen with increasing forecast horizon, indicating increasing uncertainty associated with long-term scenario estimates. Therefore, these forecasts should be interpreted as scenario estimates rather than exact predictions.

**Figure 6:**
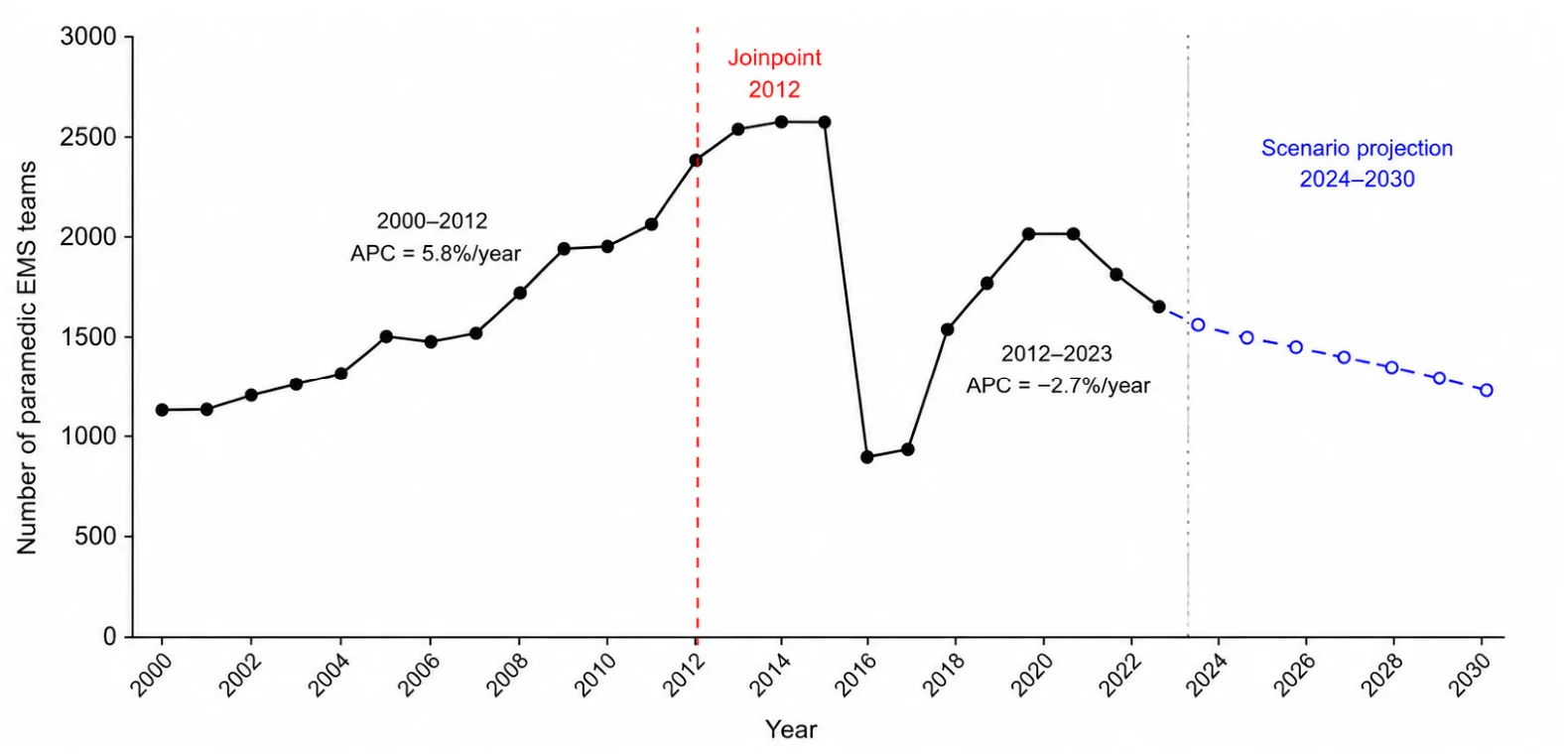
Dynamics of the number of paramedic teams in 2000–2023, results of Joinpoint analysis and scenario forecast for 2024–2030. The red dashed vertical line indicates the joinpoint identified in 2012. The blue dashed line with open circles represents the forecast scenario for 2024–2030.

The dynamics of the number of EMS paramedic brigades were characterized by a single turning point identified around 2012 (Figure 6). Between 2000 and 2012, the number of such EMS brigades increased steadily, with an average annual percentage change (APC) of 5.8% per year. The number of brigades increased from approximately 1100 in 2000 to more than 2400 by the end of the first period. After the identified turning point, the overall trend changed towards a gradual decrease (APC = −2.7% per year) between 2012 and 2023. However, the observed dynamics remained uneven, with a sharp decline in 2016–2017, a partial recovery in 2018–2021, and a subsequent decline by 2023. A scenario forecast for 2024–2030 was subsequently created in R by extrapolating the final Joinpoint segment. Assuming a continuation of the recent trend, the number of ambulance crews is projected to gradually decline from approximately 1600 in 2024 to approximately 1200–1300 by 2030. The forecast intervals gradually widen with increasing forecast horizon, indicating increasing uncertainty associated with long-term scenario estimates. Therefore, these values should be interpreted as scenario forecasts rather than exact predictions.

Although the estimated change in slope around 2012 did not reach statistical significance according to the Davis test (*p* = 0.0897), it was retained to describe the observed temporal pattern and to provide a basis for scenario forecasting.

## 4. Discussion

In 2010, the Republic of Kazakhstan experienced a sharp decline in the number of emergency medical service (EMS) stations. This may be related to the global economic crisis at the time, which impacted many countries, including Kazakhstan. Between 2008 and 2009, the country’s economy faced serious challenges, such as falling oil prices, declining budget revenues, and economic instability. In response, government spending was cut, including funding for healthcare and, in particular, the ambulance system. This could have led to the closure or reduction in stations, especially in less populated or remote areas [13].

Also, during this period, the healthcare system was reformed based on the Decree of the President of the Republic of Kazakhstan dated 13 September 2004: “State Program for Reform and Development of Healthcare in the Republic of Kazakhstan for 2005–2010” [14]. The goal of this program was to create an effective healthcare delivery system, prioritizing the development of primary health care (PHC) aimed at improving public health and preventing major socially significant diseases through “promoting the health of healthy citizens.” Budgetary funds were redistributed from the inpatient to the outpatient sector, with the widespread use of hospital-substituting technologies.

In 2017, following the issuance of Order No. 450 of the Minister of Health of the Republic of Kazakhstan dated 3 July 2017, “On Approval of the Rules for the Provision of Emergency Medical Care in the Republic of Kazakhstan,” the number of EMS stations decreased from 22 to 18. It remained at this level until 2022–2023, when it again reached 22 stations (the relative average annual increase over this period was 1.3% [CI −3.48; 6.32]). The decrease in the number of stations in 2017 may have been due to the fact that category 4 calls, which do not pose a risk to the patient’s life and health, were transferred to primary health care organizations [15].

In July 2017, Kazakhstan began reforming its emergency medical service, aimed at improving its efficiency. The main areas of change included the transition to a two-tier model for emergency care, the introduction of a differentiated call processing system, and the creation of a unified information platform for call registration, routing, and monitoring. These changes accelerated the processing of public inquiries, optimized the distribution of calls by urgency, and increased response times. At the same time, a unified national standard for ambulance equipment was introduced, in accordance with which the service’s fleet was updated [16].

The emergency medical service system in Kazakhstan retains common features characteristic of most post-Soviet countries. It is based on a centralized management model, including regional stations responsible for providing emergency medical care to the population. Regulatory requirements for the staffing and equipment of these units are largely comparable to similar requirements in other countries in the region. At the same time, the lack of qualified specialists remains one of the key problems that can reduce the level of staffing of the service and have a negative impact on the quality of medical care [17].

Pre-hospital emergency medical care for citizens of the Republic of Kazakhstan is provided by physicians (17.75%) and paramedics (82.25%) at ambulance stations. The health, and often the life, of patients depends on the knowledge and skills of medical specialists. To ensure that such knowledge and skills are of high quality and delivered in a timely manner, an effective education system is necessary. Since 2017, the Republic of Kazakhstan has begun actively training emergency medical specialists according to international standards for emergency medical care. The results of the modeling demonstrate a steady trend toward changes in the structure and functioning of the emergency medical care system in the Republic of Kazakhstan in the medium and long term. The resulting forecasts point to a further increase in the workload of the emergency medical service, consistent with global trends driven by an aging population, the increasing prevalence of chronic non-communicable diseases, and increased access to emergency medical care [9].

International experience shows that the effectiveness of the emergency medical service is largely determined not only by the number of teams and vehicles, but also by the organizational model of the service [5]. Internationally, two models of prehospital care are most common: the Franco-German and the Anglo-American. The former involves the active participation of a physician directly at the scene of the call and the implementation of the “doctor-to-patient” principle, while the latter focuses on the rapid provision of basic care by nursing staff followed by patient transportation to a specialized hospital based on the “patient-to-doctor” principle [18].

The composition of ambulance teams is determined by the urgency of the call. For less complex cases, paramedic teams are formed, which may include one paramedic and a driver, or two paramedics and a driver. A specialized medical team, designed to provide care to patients with severe and life-threatening conditions, includes a physician, a paramedic, and a driver (or a paramedic–driver) [6].

Various organizational models for emergency medical care are used worldwide. The most common model is one in which a physician is part of the mobile team along with paramedics, nurses, or other specialists. In a number of countries, the number of paramedic teams significantly exceeds the number of physician teams, with the ratio typically being approximately 3:1, and in some healthcare systems reaching 4:1 [19].

An alternative model is based on prehospital care being provided by specialists without higher medical education (paramedics). This approach is widely used in Northern and Western European countries, including Sweden, Norway, the Netherlands, Finland and the UK, where paramedics provide the bulk of emergency medical care in the pre-hospital setting. However, doctors work in the sanitary service or in a consulting working group. Compliance monitoring is based on the principle of a medical manager. In cases where the patient’s condition becomes more severe and patient management exceeds the paramedic’s capabilities, a physician consultant may be available to call and receive detailed instructions and algorithms for providing qualified medical care to a patient classified as severe or critical. This model of emergency medical care is less popular. It involves the use of paramedics only in ambulance crews in the absence of medical teams. This model is typical in Ireland and Malta [20,21,22].

Based on the data provided, it can be concluded that until 2016, the number of medical and pediatric teams remained approximately the same, while the number of paramedic teams steadily increased. In 2016, the number of both paramedic and general medical teams decreased by 2.9 times. These changes in the structure and number of ambulance teams can be explained by the healthcare reforms implemented in Kazakhstan in 2016, following the implementation of the State Program for the Development of Healthcare of the Republic of Kazakhstan “Densaulyk” for 2016–2019, which affected the organization and structure of the emergency medical care system [23]. From 2016 to 2019, the system of mobile teams at outpatient clinics was not formed; home medical care was provided by district physicians. However, during this period, the organizational basis for the subsequent formation of mobile teams was created. During the COVID-19 pandemic, mobile teams were organized to provide home care to patients. According to the Government, 3676 mobile teams were operating during the pandemic. From 2022, mobile teams handling non-urgent calls became a permanent element of outpatient services [16]. The composition of mobile teams was determined by the outpatient institution based on its resources. Indications for the dispatch of mobile teams and the procedure for their interaction with the ambulance service and the head of the emergency department were detailed. In 2024, the standard for the provision of mobile teams was defined for the first time: 1 mobile team per 25,000 assigned population [16]. According to the calculations of the Ministry of Health, approximately 647 mobile teams were functioning. The current model of mobile teams was finally formalized in 2025. According to the Ministry of Health, 580 mobile teams were operating in 2025. Thus, starting in 2020, part of the work of emergency medical teams began to be performed by mobile teams of clinics.

It should be noted that although a significant reduction in the number of medical and pediatric teams was observed in 2016, these data should be interpreted with caution. In accordance with the specified program, in 2016, the operating procedures of the ambulance service in Kazakhstan were changed, with category 4 calls (uncomplicated hypertensive crises, minor non-life-threatening injuries, elevated body temperature) being handled by mobile teams from outpatient clinics during working hours. Ambulance teams continued to respond to category 1, 2, and 3 calls 24 h a day, and also handled category 4 calls at night, on weekends, and on holidays.

In subsequent years, the number of physician teams continued to steadily decline, while the number of paramedic teams increased, reaching a peak of 2071 teams in 2021. This trend can be explained by the reform of the EMS in 2018–2020 [15], associated with a reduction in physician salaries. Furthermore, as of 1 January 2021, the primary requirement for employment as an emergency medical service physician for medical university graduates is a diploma in emergency medicine residency, despite the fact that residency in this specialty was only launched in 2019 with a three-year duration of study. These requirements, along with the insufficient number of annual residency openings in this specialty, are leading to a shortage of physicians and significantly complicating the ability to staff emergency medical service teams. Furthermore, the reduction in the number of teams may have been due to financial difficulties and budget constraints, which in turn could have led to cuts in some parts of the healthcare system. These changes were aimed at streamlining the system but could have caused temporary difficulties in providing the population with essential medical care.

An analysis of the obtained forecast data shows that the state of the emergency medical care system in Kazakhstan is characterized by a shortage of physician personnel, the need to optimize the organization of emergency services, and an increase in the number of calls that do not require direct medical intervention. Similar processes have been observed in several European countries and North America, where the expansion of paramedic competencies has ensured acceptable access to emergency care with limited human resources [24,25,26].

These results are also consistent with recent studies of the functioning of the emergency medical care service in Kazakhstan following the COVID-19 pandemic. According to a national analysis of service performance for 2019–2023, the emergency medical care system was under high pressure, especially for low-urgency calls. This indicates the need to improve triage mechanisms, develop telemedicine consultations, and strengthen the role of primary health care to reduce unnecessary burden on emergency medical care teams [5]. It should be noted that the projected reduction in the proportion of medical teams will not necessarily lead to a decrease in the quality of medical care. International experience shows that effective training for paramedics and other medical personnel, the implementation of standardized clinical protocols, and the use of modern digital technologies can maintain high survival rates and the quality of emergency care. However, the transition to a new model requires significant organizational changes, improvements to the regulatory framework, and the development of mechanisms for continuous professional training for personnel [27,28,29].

Thus, the modeling results indicate that the further development of the emergency medical care system in the Republic of Kazakhstan will focus on enhancing the role of paramedic teams, digitalizing call processing, and optimizing resource utilization. To ensure the sustainability of the system, measures are needed to strengthen the service’s staffing, implement intelligent workload forecasting systems, and develop integration between emergency medical care, primary care, and the inpatient sector. The implementation of these measures will improve the accessibility and effectiveness of emergency medical care for the population in the face of growing demographic and organizational challenges.

Strengths of the study: This study benefits from a comprehensive national longitudinal dataset collected over a 24-year timeframe (2000–2023), capturing the complete con-temporary history of emergency medicine reforms in the Republic of Kazakhstan. The inclusion of diverse metrics ranging from infrastructural station counts to localized team profiles provides a holistic view of systemic health policy impacts.

Limitations: The primary limitation rests on the statistical constraints of the long-term mathematical forecasting models. The changes observed around 2010 and 2016 may reflect structural shifts associated with health system reforms rather than gradual temporal variation. Therefore, the predictive model should be interpreted as a descriptive summary of the observed time series rather than as a definitive forecasting model.

## 5. Conclusions

The study results show a progressive annual decline in the number of emergency medical services stations over the study period. An analysis of ambulance team staffing reveals a trend toward an increase in the number of paramedics, while the number of physicians has steadily declined. A predictive model for the number of physician teams predicts a further decline through 2030, while the forecast for the number of paramedic teams remains relatively stable. This situation requires increased attention from the Ministry of Health to implement timely measures to maintain the stability and quality of emergency medical care.

## Data Availability

The raw data supporting the conclusions of this article will be made available by the authors on request.
